# Hypoxic Preconditioning Promotes the Bioactivities of Mesenchymal Stem Cells via the HIF-1α-GRP78-Akt Axis

**DOI:** 10.3390/ijms18061320

**Published:** 2017-06-21

**Authors:** Jun Hee Lee, Yeo Min Yoon, Sang Hun Lee

**Affiliations:** 1 Department of Pharmacology and Toxicology, University of Alabama at Birmingham School of Medicine, Birmingham, AL 35294, USA; j-school@hanmail.net; 2 Medical Science Research Institute, Soonchunhyang University Seoul Hospital, Seoul 336-745, Korea; yoonboo15@naver.com; 3 Departments of Biochemistry, Soonchunhyang University College of Medicine, Cheonan 330-930, Korea

**Keywords:** mesenchymal stem cell, hypoxia, 78-kDa glucose-regulated protein, cell survival, proliferation, ischemic injury

## Abstract

Mesenchymal stem cells (MSC) are ideal materials for stem cell-based therapy. As MSCs reside in hypoxic microenvironments (low oxygen tension of 1% to 7%), several studies have focused on the beneficial effects of hypoxic preconditioning on MSC survival; however, the mechanisms underlying such effects remain unclear. This study aimed to uncover the potential mechanism involving 78-kDa glucose-regulated protein (GRP78) to explain the enhanced MSC bioactivity and survival in hindlimb ischemia. Under hypoxia (2% O_2_), the expression of GRP78 was significantly increased via hypoxia-inducible factor (HIF)-1α. Hypoxia-induced GRP78 promoted the proliferation and migration potential of MSCs through the HIF-1α-GRP78-Akt signal axis. In a murine hind-limb ischemia model, hypoxic preconditioning enhanced the survival and proliferation of transplanted MSCs through suppression of the cell death signal pathway and augmentation of angiogenic cytokine secretion. These effects were regulated by GRP78. Our findings indicate that hypoxic preconditioning promotes survival, proliferation, and angiogenic cytokine secretion of MSCs via the HIF-1α-GRP78-Akt signal pathway, suggesting that hypoxia-preconditioned MSCs might provide a therapeutic strategy for MSC-based therapies and that GRP78 represents a potential target for the development of functional MSCs.

## 1. Introduction

Mesenchymal stem cells (MSCs), which have great potential for regenerative medical applications in various diseases, have gained increasing research interest over the past few decades. MSCs have been isolated from several sources, including bone marrow, adipose tissue, umbilical cord, amniotic fluid, periosteum, and fetal tissues [1]. They have the capacity to develop into bone, cartilage, muscle, marrow, tendon, adipose tissues, and vessels, depending on environmental stimuli [2]. Moreover, they regulate the immune response through various soluble factors and cell surface proteins, such as indoleamine-2,3-dioxygenase, nitric oxide, interleukin (IL)-10, and toll-like receptors [2]. Although the multitude of MSC studies have increased our understanding of MSC biology and paved the way to preclinical and clinical trials, more studies to delineate the mechanism of MSCs and to improve the efficacy of MSC therapy remain urgently needed.

Hypoxia is a crucial physiological and pathological phenomenon that regulates a wide range of cellular processes, in addition to signaling pathway transduction. In bone marrow and adipose tissues, MSCs reside in hypoxic microenvironments that have low oxygen tensions of 1% to 7% [3]. However, in the laboratory, they are commonly incubated under normoxic conditions (approximately 21% atmospheric oxygen). Conversely, in preclinical and clinical applications, MSC-based therapeutics are delivered at ischemic and hypoxic injured sites. Therefore, it has been suggested that optimization of hypoxic preconditioning for MSC expansion is important for clinical development of MSC-based therapies [4]. Accumulating evidence has shown that hypoxic preconditioning of MSCs promotes cell survival, proliferation, motility, metabolic changes, and cell retention in vitro and in vivo [5,6,7,8]. Our previous study revealed that hypoxia-preconditioned MSCs facilitate neovascularization in ischemic disease [9]. Therefore, establishing a better understanding of the mechanisms underlying MSC growth and survival under hypoxia is required for the development of MSC-based therapeutics and improvement of their efficacy.

The 78-kD glucose-regulated protein (GRP78) belongs to the heat shock protein 70 family and is widely used as a marker for endoplasmic reticulum(ER) stress [10]. GRP78 plays a key role in cell survival and apoptosis through interaction with protein kinase R-like endoplasmic reticulum kinase (PERK) [11]. Various studies have indicated that GRP78 expression is elevated during hypoxia and that GRP78 protects cardiomyocytes and neurons from hypoxia-induced apoptosis [12,13]. In addition, upregulation of GRP78 expression by physiological peptides, pharmacological agents, and gene transfer protects cardiomyocytes from hypoxic/ischemic injury [14,15,16]. However, the effect of GRP78 on hypoxia in MSCs remains elusive. In this study, we evaluated hypoxia-induced expression of GRP78 in cultured MSCs and investigated the role of GRP78 in the regulation of MSC proliferation and migration. Furthermore, we demonstrated that hypoxic preconditioning prevents cell death of transplanted MSCs in a mouse hindlimb ischemia model.

## 2. Results

### 2.1. Hypoxic Preconditioning Induces Hypoxia-Inducible Factor (HIF)-1α-Mediated 78-kDa Glucose-Regulated Protein (GRP78) Expression

To characterize MSCs under hypoxia, we conducted a long-term (10-day) survival assay. MSC survival peaked at 2 days of hypoxic stimulation (Figure S1), after which cell viability decreased in a time-dependent manner. To assess multilineage differentiation in young and senescent MSCs, multilineage differentiation capacity (osteogenic, adipogenic, and chondrogenic differentiation) was assessed in passage 4 (young) and passage 14 (senescent) MSCs (Figure S2). The differentiation capacity was higher in young than in senescent MSCs. Although reactive oxygen species (ROS) can induce cell damage, they are also key signaling molecules in stem cells for the regulation of cell functionalities such as proliferation, migration, and gene expression [17]. Thus, we measured the ROS level under hypoxic preconditioning in MSCs. ROS increased under hypoxic preconditioning (Figure S3). These results indicate that MSC functionalities might be regulated under hypoxic preconditioning.

**Figure 1:**
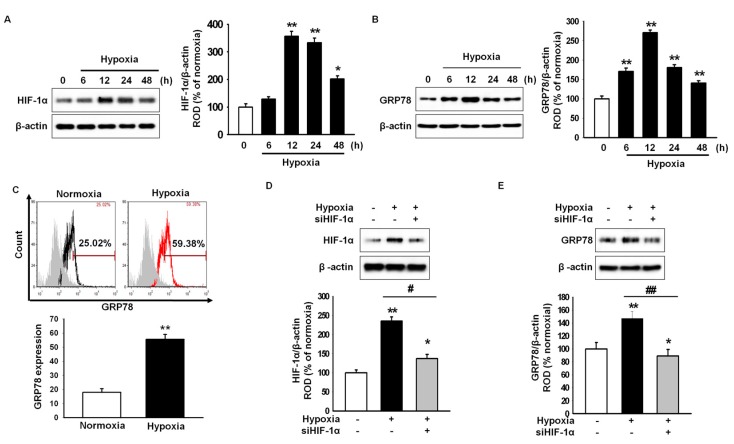
GRP78 (78-kDa glucose-regulated protein)expression is regulated by hypoxia-inducible factor (HIF)-1α in hypoxia-preconditioned mesenchymal stem cells (MSCs). (**A**) Western blot analysis of HIF-1α expression in MSCs exposed to hypoxia for 0, 6, 12, 24, or 48 h. The expression level of HIF-1α was normalized to that of β-actin. Values represent the mean ± standard errors of the means (SEM) (*n* = 3). * *p* < 0.05 and ** *p* < 0.01 vs. normoxia; (**B**) Western blot analysis of GRP78 expression in MSCs exposed to hypoxia for 0, 6, 12, 24, or 48 h. The expression level of GRP78 was normalized to that of β-actin. Values represent the mean ± SEM (*n* = 3). ** *p* < 0.01 vs. normoxia; (**C**) Flow-cytometric analysis of anti-GRP78 after exposure to normoxia or hypoxia for 12 h. The bottom panel shows standard quantification of the percentage of GRP78-positive cells. Values represent the mean ± SEM. ** *p* < 0.01 vs. normoxia (*n* = 3); (**D**) Western blot analysis of HIF-1α expression in MSCs exposed to hypoxia for 12 h after pretreatment with HIF-1α-specific siRNA (siHIF-1α). The expression level of HIF-1α was normalized to that of β-actin. Values represent the mean ± SEM (*n* = 3). * *p* < 0.05 and ** *p* < 0.01 vs. normoxia, # *p* < 0.05 vs. hypoxia; (**E**) Western blot analysis of GRP78 expression in MSCs exposed to hypoxia for 12 h after pretreatment with siHIF-1α. The expression level of GRP78 was normalized to that of β-actin. Values represent the mean ± SEM (*n* = 3). * *p* < 0.05 and ** *p* < 0.01 vs. normoxia, ## *p* < 0.01 vs. hypoxia.

To explore whether hypoxic preconditioning induces GRP78 expression in MSCs, the expression of GRP78 under hypoxia was assessed by western blot analysis. Hypoxia increased the expression of hypoxia-inducible factor 1-α (HIF-1α; Figure 1A). The level of GRP78 peaked after 12 h of hypoxic stimulation (Figure 1B). In addition, flow cytometry showed that GRP78-positive cells were significantly higher under hypoxia than under normoxia (Figure 1C). The hypoxia-induced expression of GRP78 was attenuated by HIF-1α-specific siRNAs (Figure 1D,E). These findings suggested that hypoxia induces the upregulation of GRP78 through hypoxia-mediated HIF-1α expression.

### 2.2. GRP78 Regulates Cell Cycle-Associated Proteins through the Akt Pathway

**Figure 2:**
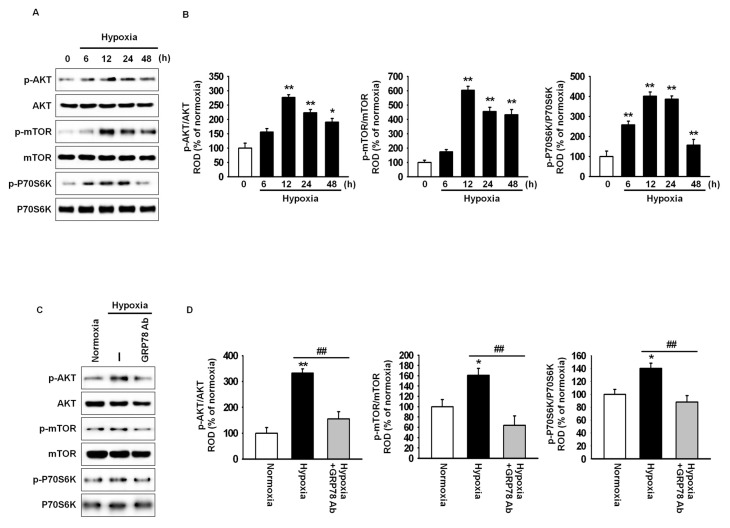
Hypoxic preconditioning enhances cell proliferation-associated signaling through the expression of GRP78. (**A**) Western blot analysis of Akt, mTOR, and p70S6k phosphorylation in MSCs exposed to hypoxia for 0, 6, 12, 24, or 48 h; (**B**) The expression levels of p-Akt, p-mTOR, and p-p70S6k were normalized to those of Akt, mTOR, and p70s6k, respectively. Values represent the mean ± SEM (*n* = 3).* *p* < 0.05 and ** *p* < 0.01 vs. normoxia; (**C**) Western blot analysis of Akt, mTOR, and p70S6k phosphorylation in MSCs exposed to hypoxia for 12 h after pretreatment with anti-GRP78 antibody (GRP78 Ab; 100 ng/mL); (**D**) The expression levels of p-Akt, p-mTOR, and p-p70S6k were normalized to those of Akt, mTOR, and p70S6k, respectively. Values represent the mean ± SEM (*n* = 3).* *p* < 0.05 and ** *p* < 0.01 vs. normoxia, ## *p* < 0.01 vs. hypoxia.

Hypoxic preconditioning enhances cell proliferation and migration through the Akt signal pathway [18]. To confirm the interaction of GRP78 with the Akt pathway in hypoxic condition, hypoxia-induced phosphorylation of Akt, mammalian target of rapamycin (mTOR), and ribosomal protein S6 kinase β-1 (also known as p70S6 kinase; p70S6k) was investigated by western blot analysis (Figure 2A). Akt, mTOR, and p70S6k phosphorylation was increased after 12 h of hypoxia, while this increase was attenuated by treatment with anti-GRP78 antibody (Figure 2A–D). These results indicated that hypoxia-induced GRP78 regulates the Akt signal pathway.

**Figure 3:**
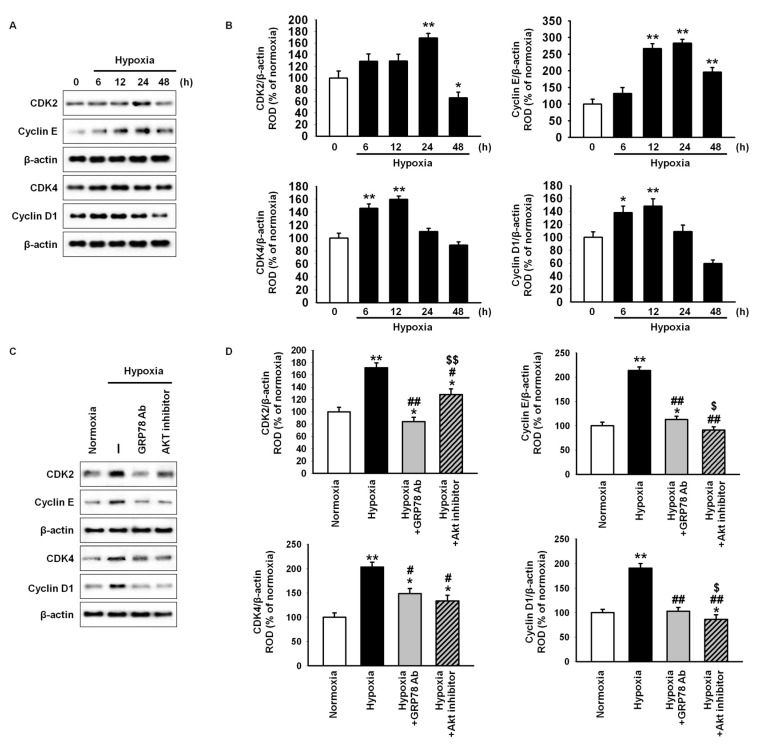
Hypoxic preconditioning increases the expression of cell cycle-associated protein through the GRP78-Akt pathway. (**A**) Western blot analysis of cyclin-dependent kinase 2 (CDK2), cyclin E, CDK4, and cyclin D1 in MSCs exposed to hypoxia for 0, 6, 12, 24, or 48 h; (**B**) The expression levels of CDK2, cyclin E, CDK4, and cyclin D1 were normalized to that of β-actin. Values represent the mean ± SEM (*n* = 3). * *p* < 0.05 and ** *p* < 0.01 vs. normoxia; (**C**) Western blot analysis of CDK2, cyclin E, CDK4, and cyclin D1 in MSCs exposed to hypoxia for 12 h after pretreatment with anti-GRP78 antibody (GRP78 Ab; 100 ng/mL) or Akt inhibitor (10^−6^ M); (**D**) The expression levels of CDK2, cyclin E, CDK4, and cyclin D1 were normalized to that of β-actin. Values represent the means ± SEM (*n* = 3).* *p* < 0.05 and ** *p* < 0.01 vs. normoxia, # *p* < 0.05 and ## *p* < 0.01 vs. hypoxia, $ *p* < 0.05 and $$ *p* < 0.01 vs. hypoxia pretreated with anti-GRP78 antibody.

To explore whether hypoxia-induced GRP78 is involved in MSC proliferation, the expression of cell cycle-associated proteins, including cyclin-dependent kinase 2 (CDK2), cyclin E, CDK4, and cyclin D1, in hypoxic condition was assessed by western blot analysis (Figure 3A). Expression of these proteins increased significantly after hypoxic stimulation (Figure 3B). Protein levels decreased significantly after treatment with anti-GRP78 antibody or Akt inhibitor (Figure 3C,D). These findings suggested that hypoxia-induced GRP78 is involved in the expression of cell cycle-associated proteins via regulation of the Akt signal pathway.

### 2.3. Hypoxia-Induced GRP78 Augments Proliferation and Migration of Mesenchymal Stem Cells (MSCs) via the GRP78-Akt Axis

**Figure 4:**
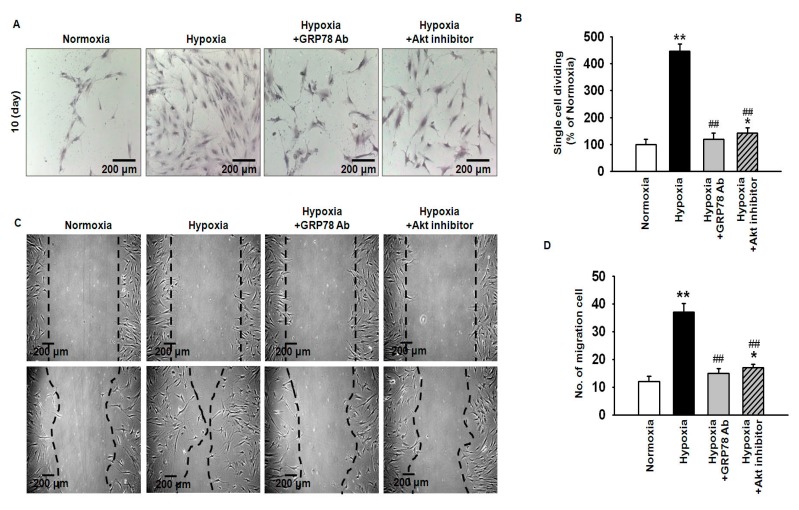
Hypoxic preconditioning augments cell proliferation and migration via GRP78-Akt axis. (**A**) Single-cell cultures of normoxic MSCs, hypoxia-preconditioned MSCs, and hypoxia-preconditioned MSCs pretreated with anti-GRP78 antibody (GRP78 Ab; 100 ng/mL) or Akt inhibitor (10^−6^ M) were stained with Giemsa after 10 days of cultivation. Scale bar = 200 μm; (**B**) Percentage of single MSCs undergoing at least one cell division after 10 days. Values represent the mean ± SEM (*n* = 3).* *p* < 0.05 and ** *p* < 0.01 vs. normoxia, ## *p* < 0.01 vs. hypoxia; (**C**) Scratch wound-healing migration assay of normoxic MSCs, hypoxia-preconditioned MSCs, and hypoxia-preconditioned MSCs pretreated with anti-GRP78 antibody (GRP78 Ab; 100 ng/mL) or Akt inhibitor (10^−6^ M). Scale bar = 200 μm; (**D**) Standard quantification of migrating cells is presented as the number of migrated cells per field. Values represent the mean ± SEM (*n* = 3).* *p* < 0.05 and ** *p* < 0.01 vs. normoxia, ## *p* < 0.01 vs. hypoxia.

To investigate the effect of hypoxia-induced GRP78 on MSC proliferation and migration, a single-cell expansion assay was conducted (Figure 4A). The results indicated that hypoxic preconditioning significantly increased MSC proliferation, while this effect was blocked by treatment with anti-GRP78 or Akt inhibitor (Figure 4B). In a wound-healing assay, migration was significantly enhanced in hypoxia-preconditioned MSCs as compared to normoxic MSCs (Figure 4C). The enhanced migration capacity was attenuated by treatment with anti-GRP78 or Akt inhibitor (Figure 4D). These results suggested that hypoxia-induced GRP78 regulates MSC proliferation and migration through activation of the Akt pathway.

### 2.4. Hypoxic Preconditioning Enhances the Survival and Proliferation of MSCs in Ischemic-Injured Tissues through Regulation of GRP78 Expression

**Figure 5:**
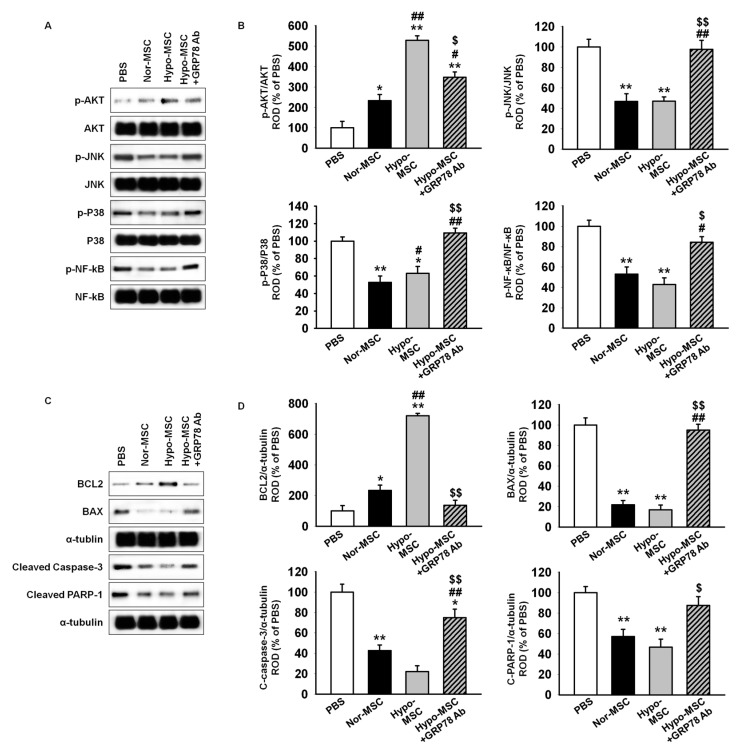
Transplantation of hypoxia-preconditioned MSCs regulates stress- and apoptosis-associated signal pathway in ischemic-injured tissue. (**A**) Western blot analysis of Akt, c-Jun N-terminal kinases (JNK), p38, and nuclear factor kappa-light-chain-enhancer of activated B cells (NF-κB) phosphorylation in ischemic sites of mice injected with phosphate buffer saline (PBS), normoxic MSCs (Nor-MSC), hypoxia-preconditioned MSCs (Hypo-MSC), and Hypo-MSCs pretreated with anti-GRP78 antibody (Hypo-MSC+GRP78 Ab; 100 ng/mL) at day 3 after MSC transplantation; (**B**) The expression levels of p-Akt, p-JNK, and p-p38, and p-NF-κB were normalized to those of Akt, JNK, and p38, and NF-κB, respectively. Values represent the mean ± SEM (*n* = 3).* *p* < 0.05 and ** *p* < 0.01 vs. PBS, # *p* < 0.05 and ## *p* < 0.01 vs. Nor-MSC, $ *p* < 0.05 and $$ *p* < 0.01 vs. Hypo-MSC; (**C**) Western blot analysis of B-cell lymphoma (BCL)2, BCL2-associated X protein (BAX), cleaved caspase-3, and cleaved poly(ADP-ribose) polymerase(PARP)-1 in ischemic sites of mice injected with PBS, Nor-MSC, Hypo-MSC, and Hypo-MSC+GRP78 Ab at day 3 after MSC transplantation; (**D**) The expression levels of BCL2, BAX, cleaved caspase-3, and cleaved PARP-1 were normalized to that of α-tubulin. Values represent the mean ± SEM (*n* = 3).* *p* < 0.05 and ** *p* < 0.01 vs. PBS and ## *p* < 0.01 vs. Nor-MSC, $ *p* < 0.05 and $$ *p* < 0.01 vs. Hypo-MSC.

To assess whether hypoxic preconditioning augments the survival and proliferation of transplanted MSCs, we generated a murine hindlimb ischemia model and transplanted MSCs in ischemic-injured sites. At postoperative day 3, ischemic-injured tissues were harvested and subjected to western blotting for stress- and apoptosis-associated proteins. Akt activation was significantly higher in lysates of tissues injected with hypoxia-preconditioned MSCs (Hypo-MSCs) than in those of tissues injected with normoxic MSCs (Nor-MSCs; Figure 5A,B). The phosphorylation of stress-associated proteins, such as c-Jun N-terminal kinases (JNK), p38, and nuclear factor kappa-light-chain-enhancer of activated B cells (NF-κB), was significantly lower in Hypo-MSCs than in Nor-MSCs (Figure 5A,B). Moreover, transplantation of Hypo-MSCs in ischemic-injured tissues activated anti-apoptosis protein, B-cell lymphoma 2 (BCL2), and suppressed pro-apoptosis proteins, including BCL2-associated X protein (BAX), cleaved caspase-3, and cleaved poly(ADP-ribose) polymerase-1 (PARP-1; Figure 5C,D). Treatment of Hypo-MSCs with anti-GRP78 antibody blocked the observed effects (Figure 5A–D).

**Figure 6:**
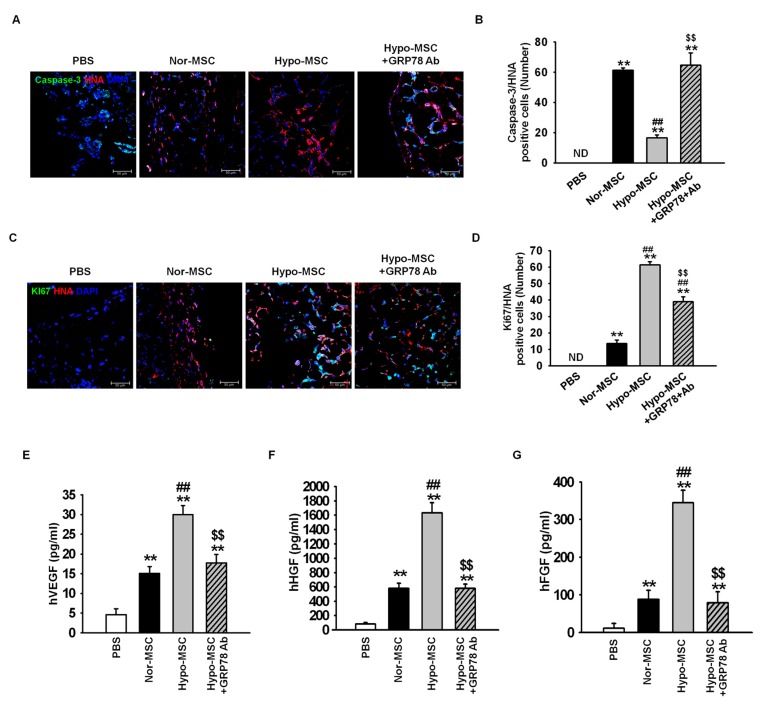
Hypoxic preconditioning facilitates survival, proliferation, and angiogenic cytokine secretion of transplanted MSCs in a murine hind-limb ischemia model. After establishing the murine hind-limb ischemia model, ischemic-injured tissues were collected at postoperative day 3. (**A**) Immunofluorescence staining for cleaved caspase-3 (green) and human nuclear antigen (HNA; red). Scale bar = 50 μm; (**B**) Apoptosis of transplanted MSCs was quantified as the number of cleaved caspase-3 and HNA double-positive cells or ND (none detected). Values represent the mean ± SEM (*n* = 3). ** *p* < 0.01 vs. PBS, ## *p* < 0.01 vs. Nor-MSC, $$ *p* < 0.01 vs. Hypo-MSCs; (**C**) Immunofluorescence staining for Ki-67 (green) and HNA (red). Scale bar = 50 μm; (**D**) Proliferation of transplanted MSCs was quantified as the number of Ki67 and HNA double-positive cells or ND. Values represent the mean ± SEM (*n* = 3). ** *p* <0.01 vs. PBS, ## *p* < 0.01 vs. Nor-MSC, $$ *p* < 0.01 vs. Hypo-MSCs; (**E**–**G**) Expression of human vascular endothelial growth factor (hVEGF) (**E**), human hepatocyte growth factor (hHGF) (**F**), and human fibroblast growth factor (hFGF) (**G**) in ischemic limb tissue lysates was determined by ELISA. Values represent the mean ± SEM (*n* = 3). ** *p* < 0.01 vs. PBS, ## *p* < 0.01 vs. Nor-MSC, $$ *p* < 0.05 vs. Hypo-MSC.

To investigate further whether hypoxic preconditioning enhances survival and proliferation of transplanted MSCs, immunofluorescence staining was used at postoperative day 3. Apoptosis of transplanted MSCs was assessed as the number of cells positive for cleaved caspase-3 and human nuclear antigen (HNA; Figure 6A). The number of apoptotic, transplanted cells was significantly lower in ischemic-injured tissues transplanted with Hypo-MSCs than in those transplanted with Nor-MSCs (Figure 6B). Proliferative transplanted MSCs in ischemic-injured tissue were identified on the basis of positive signal for both Ki67 and HNA (Figure 6C). The number of proliferative transplanted cells was significantly higher in tissues transplanted with Hypo-MSCs than in those transplanted with Nor-MSCs (Figure 6D). These effects were reversed by treatment of Hypo-MSCs with anti-GRP78 antibody (Figure 6A–D).

Finally, to determine the expression of angiogenic cytokine after transplantation of MSCs in ischemic-injured tissue, we assessed the expression of human vascular endothelial growth factor (hVEGF), human hepatocyte growth factor (hHGF), and human fibroblast growth factor (hFGF) by enzyme-linked immunosorbent assay (ELISA) at postoperative day 3. The expression levels of hVEGF, hHGF, and hFGF were significantly higher in ischemic tissue injected with Hypo-MSCs than in tissues from other groups, while this enhancement was blocked upon inhibition of GRP78 (Figure 6E–G). Taken together, these findings implied that hypoxic preconditioning promotes the survival and proliferation of transplanted MSCs in ischemic-injured tissue through GRP78-mediated suppression of stress and apoptotic signaling, and an increase in the levels of angiogenic cytokines.

## 3. Discussion

MSCs isolated from various sources, including bone marrow and adipose tissue, reside in hypoxic niches in vivo. Although oxygen concentration varies depending on the tissue type, in several tissues or organs, such as bone marrow (1–6%), adipose tissue (2–8%), brain (0.5–8%), heart (4–14%), liver (4–14%), and circulation (4–14%), they are remarkably lower than normoxia (21% O_2_) [19]. Accumulating evidence suggests that hypoxic preconditioning enhances plasticity, survival, proliferation, engraftment, and genetic stability of MSCs [4,7,19]. However, optimum oxygen tension, suitable culture time, paracrine action, and the underlying mechanism need further investigation. Our results indicated that hypoxic preconditioning (2% O_2_) promotes MSC proliferation and migration through elevated GRP78 expression. In a murine hind-limb ischemia model, hypoxic preconditioning enhanced the survival and proliferation capacity via modulation of cell-death signaling and angiogenic cytokine secretion. Moreover, our results revealed that GRP78 regulates these beneficial effects in hypoxia via the Akt signal pathway.

Hypoxia plays important roles in embryo development and in maintaining homeostasis in mammals, including humans. In hypoxic conditions, HIF-1α is the main regulator of cellular responses to hypoxia. Although the synthesis of HIF-1α is regulated by oxygen-independent mechanisms, regulation of its degradation is oxygen-dependent [20]. Under hypoxic conditions, accumulated HIF-1α is translocated into the nucleus to promote the transcription of genes involved in cell fate, metabolism, migration, invasion, metastasis, and angiogenesis [19]. Stabilized HIF-1α levels regulate various signaling molecules, such as Akt, extracellular signal-regulated kinases 1/2, JNK, p38 mitogen-activated protein kinase, NF-κB, BCL2, BAX, and other molecules in the energy metabolic pathway [21]. Furthermore, HIF-1α upregulates CXCR4, CXCR7, and CXCR1, which play pivotal roles in injured tissue-specific trafficking and homing of MSCs [18,22]. In this study, we revealed that GRP78 is regulated by the expression of HIF-1α in MSCs. GRP78 promotes cell proliferation and migration via protection against apoptosis [23]. Additionally, GRP78 promotes cell survival by protecting cells against ER stress through either repair or degradation of misfolded proteins [24]. Pharmacological reagent-mediated GRP78 expression induces cardioprotective effects against ischemic injury and oxidative stress [25]. In hematopoietic stem cells (HSCs), the GRP78 pathway regulates HSC quiescence and maintenance under hypoxia [26]. Our findings indicate that MSC proliferation and migration are regulated by GRP78 expression under hypoxic condition, suggesting that hypoxia-mediated GRP78 expression plays an important role in MSC proliferation and migration.

The serine/threonine kinase Akt is a critical signal node in several cellular stimuli [27]. In particular, phosphorylated Akt is involved in cell survival under various apoptotic stimuli, including DNA damage, nutrient deprivation, UV irradiation, cell cycle dysfunction, and chemoreagents [27,28]. Moreover, Akt signaling contributes to cell growth, proliferation, migration, metabolism, and angiogenesis [27]. Our findings confirmed that GRP78 regulates the activation of Akt, mTOR, and p70S6k under hypoxic condition. Among these signals, the expression of cell cycle-associated proteins, including CDK2, cyclin E, CDK4, and cyclin D1, was increased through the GRP78-Akt axis. In support of our results, Akt is a downstream target of GRP78 in ER stress-tolerant lung cancer cells [29]. In addition, GRP78 and Cripto complex activate phosphatidylinositol-4,5-bisphosphate 3-kinase, which acts upstream of Akt, in HSCs [26]. Moreover, hypoxic preconditioning-mediated GRP78 increased MSC migration through the regulation of Akt phosphorylation. To our knowledge, this is the first study to show that hypoxic preconditioning enhances the potential for proliferation and migration in MSCs via the HIF-1α-GRP78-Akt signal axis.

To explore the effect of hypoxic preconditioning on transplanted MSCs in ischemic-injured tissue further, a murine hind-limb ischemia model was established. Hypoxic preconditioning enhanced the survival and proliferation of transplanted MSCs through the regulation of stress- and apoptosis-associated proteins and augmentation of angiogenic cytokines, such as hVEGF, hHGF, and hFGF. These effects were regulated by HIF-1α-mediated GRP78 expression. GRP78 protects cardiomyocytes against hypoxic/ischemic injury-induced apoptosis via downregulation of caspase-3 activation [12]. High levels of GRP78 protect neural cells against ischemic injury through the regulation of autophagy [30]. Our findings demonstrated that hypoxia-preconditioned MSCs have a survival advantage in ischemic tissues controlled by GRP78, suggesting that regulation of GRP78 is important to improve the outcome of MSC transplantation for ischemic injury repair.

## 4. Materials and Methods

### 4.1. Cell Culture

Human adipose-derived MSCs obtained from the American Type Culture Collection (ATCC; Manassas, VA, USA) were used in all experiments. MSCs were free of hepatitis B virus, hepatitis C virus, human immunodeficiency virus, and syphilis, and negative for mycoplasma. The supplier certified that the MSCs expressed MSC surface markers (CD73 and CD105) and showed adipogenic and osteogenic differentiation potential when cultured with specific differentiation media. Cells were cultured in alpha-minimum essential medium with 10% (*v*/*v*) fetal bovine serum, 100 U/mL of penicillin, and 100 mg/mL streptomycin (all from Thermo-Fisher Scientific, Waltham, MA, USA). Cells were grown in a humidified incubator at 37 °C with 5% CO_2_.

### 4.2. Hypoxic Preconditioning

MSCs were incubated in a modular incubator chamber (IB Science, Daejeon, Korea) containing a gas mixture composed of 2% O_2_, 5% CO_2_, and balanced N_2_, for 0, 6, 12, 24, or 48 h, depending on the experimental condition, at 37 °C.

### 4.3. Flow Cytometry

To examine the levels of GRP78 expression under normoxic or hypoxic culture for 12 h at 37 °C, cells were harvested and stained with anti-GRP78-DyLight488 (Abcam; Cambridge, UK) and evaluated using a Cyflow Cube 8 kit (Partec, Münster, Germany). Data were analyzed using standard FSC Express software ver. 5 (De Novo Software, Los Angeles, CA, USA).

### 4.4. Western Blot Analysis

Cells and tissue homogenates were used for protein extraction in lysis buffer. Protein was quantified by bicinchoninic acid assay. Proteins (20 μg protein) were separated by 10% sodium dodecyl sulfate-polyacrylamide gel electrophoresis and transferred to nitrocellulose membranes for antibody probing. After washing with TBS-T (10 mM Tris-HCl, pH 7.6, 150 mM NaCl, 0.05% Tween-20), the membranes were blocked with 5% bovine serum albumin (BSA) in TBS-T for 1 h and then incubated overnight at 4 °C with primary antibodies specific to HIF-1α, GRP78, Akt, p-Akt, mTOR, p-mTOR, p70S6k, p-p70S6k, CDK2, cyclin E, CDK4, cyclin D1, p-JNK, p-p38, p-NF-κB, BCL2, Bax, cleaved caspase-3, cleaved PARP-1, α-tubulin, and β-actin (Santa Cruz Biotechnology, Dallas, TX, USA). After incubation of the membranes with peroxidase-conjugated secondary antibodies (Santa Cruz Biotechnology) for 1 h at room temperature, protein bands were detected using enhanced chemiluminescence reagent (Amersham Biosciences, Little Chalfont, UK) in a dark room.

### 4.5. Single-Cell Expansion Assay

Single MSCs were seeded into individual wells of a 96-well culture plate by limited-dilution assay. Cell suspensions containing 1 × 10^3^ cells in 10 mL complete medium were diluted 1:10 (cells: complete medium), and 100 μL of the dilution (~1 cell/100 μL) was seeded into the 96-well plate. The cells were then cultured under normoxic or hypoxic conditions, or under hypoxia with pretreatment with anti-GRP78 antibody (100 ng/mL) or Akt inhibitor (10^−6^ M; Sigma-Aldrich, St. Louis, MI, USA) in a humidified incubator. Each well was examined for MSC growth at day 10.

### 4.6. Scratch Wound-Healing Migration Assay

MSCs were cultured to 90% confluence in 6-well cell culture plates in 4 mL of growth medium per well. To inhibit cell proliferation, cells were treated with mytomycin C (10 μg/mL; Sigma-Aldrich) for 3 h before assay. The cell layer was scratched using a 2-mm-wide tip to make a line-shaped wound. The cells were then pretreated with anti-GRP78 antibody (100 ng/mL) or Akt inhibitor (10^−6^ M) at 37 °C, and incubated under normoxic or hypoxic condition for 12 h at 37 °C. The cells were allowed to migrate and images were acquired under an inverted microscope (Eclipse TE300; Nikon, Tokyo, Japan).

### 4.7. Murine Hind-Limb Ischemia Model and Cell Transplantation

Eight-week-old male nude BALB/c mice (Biogenomics, Seoul, Korea) maintained under a 12-h light/dark cycle were used. All animal experiments were performed in accordance with the regulations of Soonchunhyang University, Seoul Hospital. All animal procedures were approved by the Institutional Animal Care and Use Committee of Soonchunhyang University, Seoul Hospital, Korea (project number: IACUC2013-5; 6 February 2014). The murine hind-limb ischemia model was established as previously described [31], with minor modifications. Ischemia was induced by ligation of the proximal femoral artery and the boundary vessels of the mice. At no later than 3 h after surgery, PBS, Nor-MSCs, Hypo-MSCs, and Hypo-MSCs pretreated with anti-GRP78 antibody were injected intramuscularly into the ischemic thigh (5 × 10^5^ cells/100 μL PBS per mouse; five mice per treatment group). Cells were injected into five ischemic sites.

### 4.8. Immunofluorescence Staining

At postoperative day 3, ischemic-injured thigh areas were removed and fixed with 4% paraformaldehyde (Affymetrix, Santa Clara, CA, USA). Each tissue sample was embedded in paraffin and sectioned at 4-μm thickness. Immunofluorescence staining was performed using primary antibodies against anti-Ki67 (Santa Cruz Biotechnology), cleaved caspase-3 (Santa Cruz Biotechnology), human nuclear antigen (HNA; Millipore, Billerica, MA, USA), and secondary antibodies conjugated to Alexa 488 and Alexa 594 (Thermo-Fisher Scientific). Nuclei were visualized by staining with 4′,6-diaminido-2-phenylindol (Sigma-Aldrich). Stained slides were imaged by confocal microscopy (Olympus, Tokyo, Japan).

### 4.9. Determination of Human Angiogenic Cytokine

Human angiogenic cytokines released from transplanted MSCs in ischemic-injured tissue were assessed by ELISA at postoperative day 3. After quantification of the protein in ischemic-injured tissue homogenates by bicinchoninic acid (BCA) protein assay (100 μg protein), the levels of human VEGF, human FGF, and human HGF were determined using commercially available ELISA kits (R&D Systems, Minneapolis, MN, USA) according to the manufacturer’s protocol. The levels of cytokines were quantified by measuring the absorbance at 450 nm using a microplate reader (Tecan Group AG, Männedorf, Switzerland).

### 4.10. Statistical Analysis

All data are presented as means ± standard errors of the means (SEMs). All experiments were analyzed by one-way analysis of variance (ANOVA) followed by comparisons of the treatment and control groups using the Bonferroni-Dunn test. All *p*-values < 0.05 were considered statistically significant.

## 5. Conclusions

In this study, we investigated the potential of hypoxic preconditioning in MSCs to enhance the proliferation and migration potential and improvement of survival and angiogenic cytokine expression in ischemic sites. Under hypoxic preconditioning, HIF-1α-mediated GRP78 plays roles in proliferation, migration, survival, and angiogenic cytokine secretion through regulation of the Akt signal pathway. The results suggest that the regulation of GRP78 might represent a novel target for MSC-based therapies in several ischemic diseases.

## Supplementary Materials

Supplementary materials can be found at www.mdpi.com/1422-0067/18/6/1320/s1.
