# Supplementary material for: Hypoxic Preconditioning Promotes the Bioactivities of Mesenchymal Stem Cells via the HIF-1α-GRP78-Akt Axis
Source: Int J Mol Sci. 2017 Jun 21;18(6):1320. doi: 10.3390/ijms18061320 (PMC5486141; doi:10.3390/ijms18061320)
Supplement: Supplementary file 1 [file ijms-18-01320-s001.zip › ijms-184656-supplmentary.doc]

**Supplemental Materials**

**Hypoxic Preconditioning Promotes the Bioactivities of Mesenchymal Stem Cells via the HIF-1α-GRP78-Akt Axis**

Jun Hee Lee^1^, Yeo Min Yoon^2^ and Sang Hun Lee^2,3,^*

^1^Department of Pharmacology and Toxicology, University of Alabama at Birmingham School of Medicine, Birmingham, AL 35294, USA

^2^Medical Science Research Institute, Soonchunhyang University Seoul Hospital, Seoul 336-745, Republic of Korea

^3^Departments of Biochemistry, Soonchunhyang University College of Medicine, Cheonan 330-930, Korea

**Supplemental Materials and Methods**

**1. Cell viability assay**

MSCs were incubated in a 96-well plate (2×10^3^ cells/well) under hypoxia . Cell viability was determined at 2, 4, 6, 8, and 10 days using a modified 3-(4,5-dimethyl-thiazol-2-yl)-2,5-diphenyltetrazolium bromide (MTT) assay, which is based on the conversion of the tetrazolium salt 3-(4,5-dimethylthiazol-2-yl)-5-(3-carboxymethoxyphenyl)-2-(4-sulfophenyl)-2-tetrazolium to formazan by mitochondrial NAD(P)H-dependent oxidoreductase enzymes. Formazan levels were quantified by measuring the absorbance at 575 nm using a microplate reader (Tecan, Männedorf, Switzerland).

**2. Senescence-associated β-galactosidase staining assay**

Senescence was assessed by measuring the percentage of cultured cells that stained positively for senescence-associated beta-galactosidase (SA-β-gal) activity. Cells were washed with phosphate-buffered saline (PBS) twice and fixed with 2% formaldehyde/0.2% glutaraldehyde (Sigma-Aldrich, St. Louis, MI, USA). Fixed cells were incubated at 37 °C (without CO_2_) for 12 h with a β-gal staining solution (1 mg/mL X-Gal, 40 mM citric acid–sodium phosphate buffer, 150 mM NaCl, 2 mM MgCl_2_, 5 mM potassium ferrocyanide, and 5 mM potassium ferricyanide; pH 6.0; Sigma Aldrich). Stained (blue, positive) and non-stained (negative) cells were counted by using phase contrast microscopy (Nikon, Tokyo, Japan).

**3. MSC differentiation**

For MSC differentiation, cells were grown in StemPro adipogenic, osteogenic, or chondrogenic culture medium (Thermo-Fisher Scientific, Waltham, MA, USA) for 1 week, 1 week, and 2 weeks, respectively. Adipocytes were stained with oil red O (Sigma-Aldrich) for 15 min, osteoblasts with alkaline phosphatase stain kit (Sigma-Aldrich), and chondrocytes with safranin O (Sigma-Aldrich).

**4. Dihydroethidium (DHE) staining**

To measure superoxide anion levels in cultured MSCs, MSCs were incubated under hypoxia for 12 h at 37 °C. Then, the cells were washed with PBS three times, and incubated with 10 μM DHE (Sigma-Aldrich) for 30 min at 37 °C. After washing with PBS three times, samples were visualized by flow cytometry (Cyflow Cube 8 FACS; Sysmex Partec, Görlitz, Germany). Data were analyzed using standard FSC Express software (De Novo Software).

**Supplemental Figure Legends**

**Figure S1.** Effect of hypoxia on survival in MSCs.The survival of MSCs was assessed by MTT assay under hypoxia for 0, 2, 4, 6, 8, and 10 days. Values represent the mean ± SEM (*n* = 3). * *p* < 0.05 and ** *p* < 0.01 vs. normoxia (white bar).

**Figure S2.** Assessment of differentiation capacity in senescent MSCs.(**A**) Cellular senescence was assessed with senescence-associated β-galactosidase (SA-β-gal) staining in passage 4 (P4) and 14 (P14) MSCs. MSC senescence was quantified as the number of SA-β-gal-positive cells. Scale bar = 200 μm. Values represent the mean ± SEM (n = 3). ***p* < 0.01 vs. P4 MSC; (**B**) P4 and P14 MSCs were differentiated into osteocytes, adipocytes, and chondrocytes. Osteogenic, adipogenic, and chondrogenic differentiation was assessed by alkaline phosphatase, oil red O, and safranin O staining, respectively. Scale bar = 200 μm.

**Figure S3.** Generation of ROS in MSCs under hypoxic preconditioning.The level of cellular ROS was assessed using flow cytometry of DHE-stained cells. ROS generation was quantified as the percentage of DHE-positive cells. Values represent the mean ± SEM (*n* = 3). ** *p* < 0.01 vs. control.
